# Dynamics of Graft Function Measured by DNA-Technology in a Patient with Severe Aplastic Anemia and Repeated Stem Cell Transplantation

**DOI:** 10.1155/2014/576373

**Published:** 2014-03-04

**Authors:** Anna Karastaneva, Christian Urban, Herwig Lackner, Wolfgang Schwinger

**Affiliations:** ^1^Department of Pediatrics/Pediatric Hematology and Oncology Unit, Medical University Plovdiv, 15A Vasil Aprilov Boulevard, 4000 Plovdiv, Bulgaria; ^2^Division of Pediatric Hematology and Oncology, Department of Pediatrics and Adolescent Medicine, Medical University Graz, Auenbruggerplatz 34/2, 8036 Graz, Austria

## Abstract

Although bone marrow transplantation (BMT) from an HLA identical sibling is considered as treatment of choice in pediatric patients with severe aplastic anemia (SAA), a significant number of them experience graft failure (GF) after BMT. We report a case of an 8-year-old male patient with SAA who presented with a complicated posttransplant course due to parvovirus B19 infection and GF. A subsequent attempt to support the graft by antithymocyte globulin (ATG) and a peripheral stem cell boost resulted in transitory autologous recovery of hematopoiesis followed by mixed chimerism, supported by donor lymphocyte infusions (DLIs) and finally graft rejection with relapse of SAA. Permanent complete chimerism was achieved by a second BMT. Dynamics of graft function, measured by a single nucleotide polymorphism (SNPs) analysis, are discussed.

## 1. Introduction

Severe aplastic anemia (SAA) is a rare life-threatening disease characterized by pancytopenia and marked bone marrow hypocellularity. The diagnostic criteria for SAA include bone marrow hypocellularity to less than 25% and two of the following: absolute neutrophil count (ANC) <0.5 × 10^9^/L, platelets <20 × 10^9^/L, and reticulocyte count <40 × 10^9^/L [1]. The bone marrow failure is most likely attributed to immune-mediated mechanisms resulting in activation of cytotoxic lymphocytes which eventually induce apoptosis [2] and severe reduction of hematopoietic progenitors. The causative agents of enhanced lymphocyte activation remain unclear.

First-line treatment of SAA in pediatric patients is bone marrow transplantation (BMT) from an HLA identical sibling. Alternatively, for patients lacking an HLA-matched related donor, immunosuppressive therapy with an ATG-based regimen is recommended. Although BMT is considered as treatment of choice, a significant number of patients experience early or late graft failure (GF) after BMT. Analysis of chimerism is an important tool for evaluation of graft function, early detection of GF, and identification of its possible causes in the posttransplant period. Single nucleotide polymorphism- (SNP-) based assays represent a novel rapid method for assessment of hematopoietic chimerism after allogeneic BMT [3].

## 2. Material and Methods

Quantitative analysis of donor chimerism from peripheral blood was performed by an RT-PCR, based on single nucleotide polymorphisms (SNPs) assessment. Informative SNPs are identified and used as a tool to distinguish between donor/recipient cells after BMT. All SNPs from the peripheral blood samples of donor and recipient were compared before BMT. In the following analysis at least 2 informative SNPs were selected. After BMT cell subpopulations were isolated from the recipient's peripheral blood sample, using Miltenyi immune magnetic method (MACS). The purity of isolated cell subtypes was assessed by a fluorescence-activated cell sorting (FACS) analysis. DNA was extracted from the basic material and the selected subpopulations and RT-PCR was performed for measuring the percentage of chimerism [3].

## 3. Case Report

A previously healthy 8-year-old boy was transferred to the Division of Pediatric Hematology and Oncology in Graz in February 2011 with a one-month history of pancytopenia preceded by a febrile episode. Clinical course, laboratory findings, and bone marrow aspiration were highly suggestive of idiopathic aplastic anemia. Prior to transfer the patient had received 3 erythrocytes and 7 platelet transfusions.

Physical examination revealed pallor and hematomas on the lower extremities. Blood cell counts were ANC, 0.24 × 10^9^/L; reticulocytes, 18 × 10^9^/L; platelets, 17 × 10^9^/L. Parvovirus B 19 (PVB19) PCR was tested positive above detection limit in bone marrow. Bone marrow aspiration and biopsy confirmed marked hypocellularity without signs of malignant infiltration, fibrosis, or dysplastic changes. There was no evidence of chromosomal abnormalities associated with MDS. The patient fulfilled all criteria for SAA and was prepared for allogeneic bone marrow transplantation from his HLA-identical brother (BMT1).

**Figure 1:**
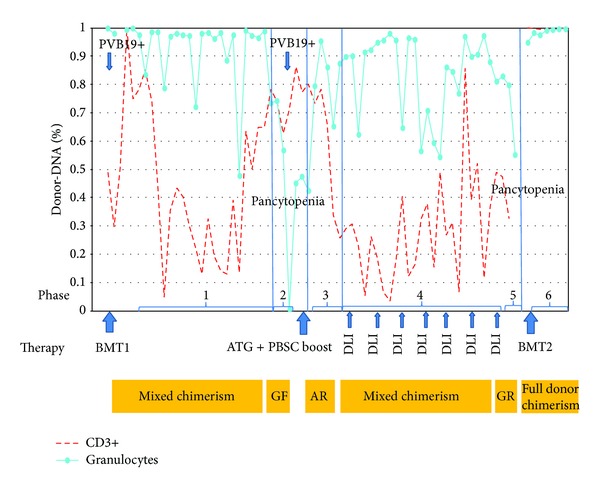
Donor chimerism analysis and graft function between BMT1 and BMT2. Phase 1—mixed chimerism; phase 2—graft dysfunction due to PVB19 reactivation: increasing donor T-cells and declining donor granulocytes; phase 3—autologous recovery after ATG + PBSC boost: declining donor T-cells and donor granulocytes, but stable peripheral blood counts; phase 4—mixed chimerism after PBSC boost with DLI support; phase 5—graft rejection: declining donor T-cells and granulocytes, pancytopenia; phase 6—stable T-cell and granulocyte engraftment after BMT2; BMT1: first bone marrow transplantation; BMT2: second bone marrow transplantation; ATG: antithymocyte globulin; PBSC boost: peripheral blood stem cell boost; DLI: donor lymphocyte infusion; GF: graft failure/dysfunction; GR: graft rejection; AR: autologous recovery; PVB19+: PCR-PVB19 (BM) positivity.

The conditioning regimen consisted of cyclophosphamide (4 × 50 mg/kg/d) and rabbit ATG (3 × 20 mg/kg/d). A bone marrow suspension of 1100 mL containing 3.53 × 10^8^/kg nucleated cells with 4.1 × 10^6^ CD34+/kg and 0.29 × 10^8^ CD3+/kg was administered. After transplantation immunosuppression was started with cyclosporine A. WBC engraftment followed on day +25 with counts exceeding 1.0 × 10^9^/L. Platelets were above 50 × 10^9^/L after day +23. The last red blood cell transfusion was administered on day +26. The immunosuppressive therapy was discontinued 4 months after BMT1. In the follow-up period SNP analysis revealed mixed chimerism (Figure 1, phase 1), but peripheral blood counts continued to improve. However, pancytopenia reoccurred 18 months after BMT1. The boy was subfebrile and pale, with hematomas on the lower extremities. Inflammatory parameters were within normal ranges. Bone marrow PCR, however, was again positive for parvovirus B 19. Chimerism analysis revealed increasing donor T-cell content and in parallel declining percentage of donor granulocytes (Figure 1, phase 2).

Due to possible reactivation of parvovirus B19-infection and the presence of circular exanthema on the lower extremities, therapy with immunoglobulin was administered and an attempt to enhance the graft by a stem cell boost from peripheral blood of the donor was undertaken. The preparative regimen with horse ATG (40 mg/kg/d) for 4 days was well tolerated by the patient. The mononuclear cell suspension administered was T-cell depleted by MACS cell separation and consisted of a pure fraction of 10 × 10^6^/kg CD34+ cells. Prophylactic immunosuppressive therapy with mycophenolate mofetil was given for 17 days. No signs of GVHD were observed.

WBC increased up to 17.24 × 10^9^/L on day +14. Platelet counts continued to increase progressively. Chimerism analysis of donor granulocytes demonstrated slowly increasing donor DNA to a maximum of 93.98% in the immediate period after the PBSC boost and a subsequent decrease in the percentage of both donor-derived granulocytes and lymphocytes while peripheral blood counts were increasing. These findings were interpreted as an imminent loss of graft function and partial recovery of autologous hematopoiesis (Figure 1, phase 3). Attempts to restore donor chimerism by donor lymphocyte infusions (DLI) within the following months resulted in establishment of a state of mixed donor/recipient chimerism (Figure 1, phase 4). The patient maintained near normal peripheral blood counts for 4.5 months when pancytopenia reoccurred. SNP analysis demonstrated a decline in both donor granulocyte and T-cell fractions, suggesting graft rejection (Figure 1, phase 5). As the previous attempt to support graft function with a T-cell depleted PBSC boost and repetitive DLIs was unsuccessful, the patient was scheduled for a second BMT (BMT2). Conditioning with thiotepa/fludarabin/cyclophosphamide/ATG was initiated. The graft consisted of 3.19 × 10^8^ nucleated cells/kg and 4 × 10^6^ CD34+ cells/kg. During the entire follow-up period of 6.5 months stable chimerism was maintained with peripheral blood cell counts within normal ranges (Figure 1, phase 6). However, despite the immunosuppressive therapy, the patient developed chronic GVHD of the skin followed by liver involvement after day +142 after BMT2.

## 4. Discussion

GF after HLA-matched sibling transplantations for SAA remains a major clinical problem and its incidence ranges widely from 5 to 50% [4–6]. Late GF is defined as ANC < 500/*μ*L for at least 14 days after initial engraftment [6]. It occurs most often within the first 100 days after BMT, but cases as late as 10 years after transplantation are also described [7–9]. GF can occur either due to graft rejection (GR) or graft dysfunction. The mechanism of GR is considered a result of regrowth of host immunocompetent cells, mostly a population of host T-cells, and simultaneous loss of donor cells [9, 10]. Factors contributing to GR include (1) prior multiple blood transfusions, (2) low number of cells infused, (3) degree of HLA disparity, (4) T-cell depletion, and (5) type of immunosuppression used [6, 9, 10]. Potential causes of graft dysfunction without evidence of host immunologic reaction against the graft are low stem cell inoculum, posttransplant viral infection, and drug toxicity [10]. SNP analysis was used to visualize white blood cell as well as T-cell, B-cell, and granulocyte chimerism on a regular weekly basis. A close monitoring was necessary to be able to interfere early in case of switch of chimerism. Since T-cells were almost 90% of donor origin GR was excluded as a reason for the first GF in our case. It is supposed that parvovirus B19 detected by PCR at that time led to donor T-cell activation and suppression of donor hematopoiesis.

The presence of parvovirus B 19 viraemia, confirmed by PCR, both at diagnosis and relapse, suggests PVB19 as a possible causative agent of SAA and GF 18 months after BMT1 in our case. Virus monitoring showed no evidence of parvovirus infection in the interval between the diagnosis and GF. There are very few published data reporting parvovirus B 19 as a pathogen, associated with SAA in immunocompetent and otherwise healthy individuals [11–14], while it is known to induce prolonged bone marrow aplasia in immunosuppressed patients [15, 16]. Although the connection between SAA and viral infections (hepatitis viruses, EBV, CMV, parvovirus B 19, HHV-6, and HIV) has not yet been reliably documented, direct effect or immune-mediated mechanisms of bone marrow failure are discussed [11, 17]. An attempt to clarify the role of parvovirus B 19 in SAA was made by Qian et al. in 2002 [18]. They tested sera of 30 pediatric patients with SAA and 30 healthy controls for parvovirus B 19 viraemia using IgM-specific antibodies or PCR. A significantly higher frequency of PVB19 viraemia was detected in patients with SAA, 20% versus 0%, respectively [18]. Another study from 2005 proved PVB19 viraemia in 40.7% of 27 SAA patients in contrast to 5% of the control group (*n* = 20) [19].

In our patient parvovirus B19-PCR was positive at diagnosis without any preceding history of immune deficiency or chronic hemolytic anaemia. At the time of reactivation, the patient was also considered to be immunocompetent as immunologic recovery was already achieved. A previous report with parvovirus B 19 associated SAA encompasses another 3 patients who underwent either matched sibling donor BMT (2 patients) or matched unrelated donor PSCT (1 patient) [20]. No complications during the follow-up period of these patients occurred, although PVB 19 is generally considered a risk factor for graft dysfunction in HSCT recipients [20].

Treatment alternatives after graft failure in patients with SAA include administration of rhGM-CSF, second BMT (BMT2) from the same or alternative donor, and allogeneic peripheral blood stem cell (PBSC) rescue [6, 21, 22].

The rationale for rhGM-CSF use after GF is possible acceleration of trilineage hematopoiesis and/or prevention of lethal infectious complications, thus increasing the time span for hematopoietic recovery [22]. This option is associated with poor response rates and recurrent severe pancytopenia shortly after discontinuation of rhGM-CSF [6]. Second HLA-matched BMT from the previously used donor is often compromised by significant morbidity and mortality, mainly due to infectious causes or other serious complications such as GVHD [6, 8]. De Medeiros et al. report an overall survival after BMT2 of 50% for patients with primary and secondary GF, with a significant advantage of patients with late graft failure (60% to 17%, resp.) [8]. Several factors have been established as predictors for favorable outcome after a reinfusion of donor BM. In general, transplant-related mortality is inversely proportional to time interval between initial and subsequent transplantations [9]. Different intervals between BMT1 and GF are identified as prognostic of a favorable outcome—60 days, 80 days, 90 days, and 6 months [5, 8, 9, 23, 24]. Age of recipient at BMT2 <10 years is also associated with favorable prognosis [5].

Although our patient fulfilled all criteria for favorable outcome after BMT2, a T-cell depleted PBSC boost from the same donor following treatment with ATG was the preferred therapeutic approach aiming at enhancement of graft function and rapid hematologic recovery while minimizing the risk of serious complications such as GVHD [6]. However, the T-cell depleted graft could not be sustained, but pretreatment with ATG probably resulted in transient recovery of autologous hematopoiesis, as it is known for patients treated with ATG without a donor [25]. However, neither AR nor fully functioning donor hematopoiesis could be sustained by repetitive DLIs and only a second BMT resulted in permanent full donor chimerism.
